# Informal Palliative Care at Home: A Focus Group Study Among Professionals Working in Palliative Care in Portugal

**DOI:** 10.3390/healthcare13090978

**Published:** 2025-04-23

**Authors:** Vanessa Marrazes, Laura Gonçalves, Ana Querido, Carlos Laranjeira

**Affiliations:** 1 School of Health Sciences, Campus 2, Polytechnic University of Leiria, Morro do Lena, Alto do Vieiro, Apartado 4137, 2411-901 Leiria, Portugal; vanessambmarrazes@gmail.com (V.M.); ana.querido@ipleiria.pt (A.Q.); 2 Inpatient Palliative Care Unit, Local Health Unite of Leiria Region, Rua do Hospital 7, 2460-051 Alcobaça, Portugal; laurinha.lcg@gmail.com; 3 Centre for Innovative Care and Health Technology (ciTechCare), Polytechnic University of Leiria, Campus 5, Rua das Olhalvas, 2414-016 Leiria, Portugal; 4 RISE Health Research Group, University of Porto, 4200-450 Porto, Portugal; 5 Comprehensive Health Research Centre (CHRC), University of Évora, 7000-801 Évora, Portugal

**Keywords:** informal caregivers, home-based care, preparedness, palliative care, qualitative study

## Abstract

**Background/Objectives:** Informal caregivers (ICs) are the backbone of home-based palliative care (PC) because they play a vital role in offering more seamless and timely support, preventing suffering and fostering comfort and dignity. Specialized home-based PC teams must recognize ICs’ unmet needs in fulfilling daily responsibilities and enhance their preparedness for caring. In this vein, this study aimed to carry out the following: (a) explore how PC professionals perceive the preparedness of ICs to provide PC at home and (b) determine what barriers and enablers they consider essential for the delivery of high-quality PC at home. **Methods:** Using purposeful sampling, thirty-four professionals from four disciplines who worked in specialized PC teams were recruited for a descriptive qualitative research study. Four focus group discussions were conducted from September 2024 to January 2025. This was followed by a thematic analysis according to Braun and Clarke’s framework. The findings are reported following the COREQ checklist. **Results:** Most of the participants were female nurses (*n* = 22) with a mean age of 41.8 ± 8.62 years. Three overarching themes were identified: (1) IC needs and motivations for providing care; (2) perceived barriers to good-quality palliative home care; and (3) perceived enablers of good-quality palliative home care. The IC’s preparedness depends on personal characteristics, health status, the scope of tasks, and the ramifications stemming from the complexity of the illness trajectory. **Conclusions:** Professionals deemed it essential for PC staff to be experienced, accessible, person-centered, and proactive. Furthermore, effective communication and a well-defined network for requesting certain community resources or services were deemed crucial for delivering high-quality PC at home.

## 1. Introduction

Population aging is a growing trend observed globally, especially in developed countries, where an increase in average life expectancy has been observed in recent decades [1]. In addition to aging, an exponential increase in chronic diseases has also imposed pressures and countless challenges for both people and health systems [2]. Given this scenario, the provision of palliative care (PC) has become a priority for the global agendas of decision-makers as a response to the growing and highly complex needs presented by patients with palliative needs and their families [3].

Health systems still lack primary healthcare policies, such as home PC, which increase health expenditure and the use of hospital services in terms of hospitalizations and visits to emergency services [3,4,5,6]. The World Health Organization [7] estimates that, worldwide, 56.8 million people need PC, of whom 78% live in low- and middle-income countries [8]. In fact, globally, only 14% of them have access to this care [7]. Additionally, people living in rural and remote areas have limited access to PC [9]. In Portugal, as in other European countries, there has been a constant increase in the supply of specialized PC services, ranking the country among the most successful in meeting the goals established by the European Association for Palliative Care (EAPC) [10]. However, current PC models are still insufficiently adapted to specific territorial characteristics, which highlights the need to invest in PC in the community and in promoting health literacy [11,12,13]. Specialist community PC services are a cost-effective alternative to hospital-based care, enhancing outcomes for patients and their informal caregivers (ICs) through greater communication and superior symptom management [13,14].

According to the International Association of Palliative Care and Hospice, PC as holistic active care plays an essential role in improving the quality of life (QoL) of people with chronic and progressive diseases, as well as their ICs, by continuous monitoring throughout the disease trajectory, especially in the final phase of life [15]. In the context of PC, ICs encompass spouses, family members, friends, and neighbors, who offer unpaid essential care to ill people. However, ICs sometimes lack sufficient training to competently accomplish caregiving responsibilities, particularly during the transition from hospital to home [12,16]. As a result, ICs often feel unprepared to provide care for individuals with palliative needs living at home [17,18,19]. Numerous obstacles were identified during the transfer of patients from acute care to home-based palliative care (HPC), including physical discomfort, medication-related confusion, uncertainty over healthcare responsibilities, and emotional distress [20,21]. The lack of IC readiness may result in unexpected hospital admissions, imposing a considerable financial strain on the healthcare system, posing serious health risks for patients, and increasing the burden and exhaustion of ICs [22,23]. ICs find purpose in their duties, which drive them to adeptly navigate the challenges of caring for an individual in need [22]. To mitigate the challenges of caregiving and enable the maintenance of a meaningful life, both inside and outside caregiving duties, additional support is essential. Research has identified numerous critical aspects associated with providing PC at home, including individualized person-centered care, IC support, healthcare accessibility, multidisciplinary care delivery, and quality enhancement [24,25].

Additionally, scientific evidence suggests that the home is the preferred context for patients and their ICs. Preference rates for EoL care in the home vary between 11% and 89%; as the preferred place of death, rates vary between 51% and 55% [26]. The home is recognized as a familiar and meaningful space, which contributes to the QoL, comfort and dignity of patients and their ICs [19,27]. The WHO [28] highlights that one of the main objectives of PC is to meet the needs of ICs, as they play a central role in providing care at home. These needs, together with the insufficient responses of health systems, suggest that it is necessary to develop approaches or initiatives that empower ICs and improve the quality of the HPC approach [29].

Due to the rising prevalence of chronic illnesses, it is essential to prioritize the preparation and support of those assuming caring responsibilities [30]. Caregiving has been associated with various outcomes, including positive effects (such as personal development) and negative effects (such as emotional weariness) [31]. Previous research has indicated that those in caregiving roles are more prone to accidents and illnesses, including anxiety and depression [32,33]. Certain individuals may encounter IC load due to the escalating complexity of specific chronic illnesses. The transition to a rigorous caring role, involving assistance with daily living activities, was found to correlate with a decline in IC functionality [34]. Individuals may experience differing degrees of burden based on the nature and frequency of their caregiving duties, as well as their perceptions of the activities and problems involved in providing care [27].

The adoption of a person-centered approach that underscores the multifaceted needs of individuals, as opposed to merely addressing the physical dimensions of care, has yet to be fully implemented in primary care settings [35]. Research reveals deficiencies in communication among different healthcare professionals, a fragmentation of care, and lack of care coordination [36], hindering efficient referral systems [37]. The model of PC organization in Portugal [38] encompasses inpatient PC units; Hospital Support Palliative Care Teams that provide guidance and assistance in the hospital; and Home Palliative Care Teams (HPCT) that deliver care to patients at home and support to their families and ICs. Additionally, HPCTs provide guidance to family physicians and nurses delivering home care. This model underscores the values of accessibility and equity. However, there is an evident need for expansion, particularly in home-based palliative care. Financial and structural constraints, along with diminished political prioritization, have resulted in disparities in primary care access, attributable to regional factors and insufficient public awareness [11].

Currently, there is scant research in Portugal on the readiness of carers for informal PC provision at home, research that might offer insights into the essential elements for high-quality PC. This gap hinders the advancement of initiatives that could assist ICs. Hence, conducting interviews with PC professionals may provide more accurate insights and understanding of these elements. Furthermore, to support the implementation of the model of PC organization in Portugal, it is also important to understand the breadth of barriers and enablers to high-quality PC provision at home. Therefore, this study aims to carry out the following: (a) explore how PC professionals perceive the preparedness of ICs to provide PC at home and (b) determine what barriers and enablers they consider essential for the delivery of high-quality PC at home. In this way, we hope to contribute to a better understanding of IC needs at home in order to mitigate familial burnout and enable ICs to care for their loved ones at home.

## 2. Materials and Methods

### 2.1. Study Design

This qualitative exploratory study used a descriptive approach [39]. The study is based on critical realism theory, which recognizes the existence of different layers of reality and that one’s personal narrative and background can determine one’s experience [40]. The data were collected through focus group discussions (FGDs). A key benefit of FGDs is that interactions among participants can yield insights into diverse opinions, perceptions, or emotions regarding a specific issue, practice, or idea, which would be less attainable through individual interviews [41,42]. Moreover, FGDs can discern the elements that shape ideas, actions, or motivations within a collective framework. They promote a more authentic atmosphere, where members both influence and are impacted by one another, mirroring real-life contexts [42].

This study was reported following the Consolidated Standards for Reporting Qualitative Research (COREQ) checklist [43] (Supplementary Table S1).

### 2.2. Setting and Recruitment

Four FGDs were organized comprising nurses, doctors, psychologists, and social workers from three specialized PC teams in two Local Health Units (LHUs), in the central and southern regions of Portugal. Each FGD comprised professionals employed at the same LHU, with participation ranging from six to twelve participants (a total of thirty-four). A purposive sampling technique [44] was used to select participants based on a set of eligibility criteria. Inclusion criteria included (1) professionals working in PC with adult patients and (2) more than six months of experience in the PC area. All participants who were absent from work due to vacation or medical leave were excluded. The application of eligibility criteria was assessed by the principal researcher (V.M.). To obtain maximum sample variation, no restrictions were imposed on sex, age and professional seniority. We employed a criterion of data saturation to ascertain the number of participants for this investigation. Recruitment ceased when the inclusion of more participants or data ceased to provide significant insights [45].

### 2.3. Data Collection

The data were gathered via in-person FGDs from September 2024 to January 2025. Potential participants were initially approached by the person responsible for each LHU. The primary author (V.M.) subsequently contacted participants through email, furnishing them with comprehensive details regarding the study and scheduling the FGD, including the time and location. Lastly, a written agreement was secured from participants who consented to participate.

A moderator (V.M.) and an assistant moderator (L.G.) facilitated four FGDs in a meeting room that was suitable for the study. The participants were arranged around a table to signify the equal significance of each individual’s contributions. The FGD lasted 40 to 90 min (an average of 60 min). The moderator presented the topic and urged the attendees to openly share their experiences while underscoring the significance of anonymity and mutual tolerance for differing perspectives, including the stipulation that remarks made in the room should be confidential. Emphasis was also given to posing straightforward, open-ended, and unambiguous questions [42]. The initial open questions posed in each FGD were “Can you tell us about your experiences related to informal caregivers’ preparedness in providing PC at home?” and “What matters most for good quality PC at home?”.

The moderator guided the talks to ensure relevant subjects were addressed and all participants were prompted to contribute. Examples of prompts utilized to elicit further information and explain perspectives included “Could you provide an example?”, “Please elaborate on your meaning”, and “Elaborate further”. Identical questioning was employed across all FGDs. The participants actively engaged in talks, occasionally expressing their opinions in incomplete phrases that were then finished or elaborated upon by others. The ambiance was uplifting, characterized by fun and laughing.

Following the guidance of Krueger and Casey [42], the assistant moderator documented field notes during the interviews to thoroughly capture themes, major points, and pertinent comments. Furthermore, the field notes were employed to document non-verbal behavior and to distinguish between speakers and their tone within the group. Towards the conclusion of the discussion, the assistant moderator delivered a concise overview of the salient points articulated and prompted the participants to contribute additional remarks by posing a final, open-ended inquiry: “Is there anything else we should include?” This question is significant as it can provoke other crucial discussion points [46].

All FGDs were audio-recorded and transcribed verbatim by the main author, immediately following each session. All FGD transcriptions, in European Portuguese, were translated and then back-translated to ensure the original meaning was preserved.

### 2.4. Data Analysis

The data were analyzed following Braun and Clarke’s reflexive thematic analysis (Table 1), which is composed of a six-phase coding framework [47,48,49]. All interview transcripts were coded by the first and last author using WebQDA software (Version 3.0, Universidade de Aveiro, Aveiro, Portugal). Based on the seminal work of the authors, each transcription was meticulously analyzed and initial coding used terms from the text. Codes were classified into topics and adjusted after each interview transcript. Following the completion of interviews, the coding procedure evolved to encompass longer phrases comprising several sentences. An effort was undertaken to shift from descriptive codes to interpretive codes, identifying the broader relationships among participants’ experiences. After each interview, the coding matrix was meticulously assessed to discern the salient aspects of the participants’ experiences [45]. A hybrid approach was employed, including both deductive and inductive coding strategies [47,48]. Specific statements about the preparedness of ICs to provide PC at home and the barriers and enablers of good-quality palliative home care were generated from the quotes.

### 2.5. Trustworthiness

We utilized Guba and Lincoln’s criteria for trustworthiness [50]. Initially, we established credibility through investigator triangulation and the collection of qualitative data from many sources, including interview transcriptions, data quotes, and field notes. To bolster the validity of the findings and foster their trustworthiness, we maintained an unbiased disposition, acknowledged personal conceptions, and engaged in self-reflection during the study trajectory. Peer debriefing allows researchers to document their thoughts, biases, and reflections, thereby enhancing transparency and minimizing subjectivity. Then, we attained transferability by offering detailed descriptions of each phase of the study process. Moreover, we assured reliability by subjecting our work to an audit trail performed by independent researchers. Finally, we achieved confirmability by reflexive journaling, recording reflexive reports throughout the data collecting and analysis.

The research team employed an inductive methodology to develop thematic groupings. Multiple research sessions were held to enable the exchange and comparison of findings, along with the recognition of common themes. In the presence of divergent perspectives, a consensus-based conclusion was reached. All participants in this study are professionals working in PC with varied viewpoints and direct experiences in the domain of EoL care. The principal investigator (V.M.) is a female nurse working in the community and has practical expertise with persons with PC needs. The second researcher is a specialized mental health nurse with clinical experience in inpatient PC. The other researchers have competence in conducting research in the field of PC, as well as proficiency in qualitative inquiries (C.L. and A.Q.). These varied experiences enriched the understanding of the material and mitigated any potential biases.

### 2.6. Ethics

This research was conducted in accordance with the Declaration of Helsinki and the European General Data Protection Regulation (GDPR2016/679). The Ethics Committee of ULS of Baixo Alentejo (number: 7.4.1.) and ULS region of Leiria (41/CECHL/2023) granted permission. Prior to data collection, all potential participants were apprised of the study’s aims and the option to withdraw from the study without repercussions, both orally and in writing. Consenting participants signed informed consent forms. No financial incentives were linked with participation. The participants’ personal data were anonymized in the material, and access was restricted solely to the writers. All obtained data were stored in a digital folder with access restricted to the lead investigator. Archives will be destroyed one year after the study’s conclusion. To safeguard the privacy and consensus among participants, alpha-numeric codes (FGD1, FGD2, etc.) were associated with data quotes.

## 3. Results

### 3.1. Sample Description

In total, 34 professionals who work in PC participated in the study. Different professions composed each group to foster positive discussions safeguarding a secure and open environment. None of the participants dropped out during the interview sessions. Thirty were female, with a mean age of 41.8 ± 8.62 years (range of 27 to 63 years). The main participants in this study were nurses (*n* = 22), doctors (*n* = 6), psychologists (*n* = 4) and social workers (*n* = 2). Most of the participants have advanced training in PC (*n* = 19). In terms of years of professional experience in PC, the mean was 12.0 ± 6.7 years. The professional background of the participants is provided in Table 2.

### 3.2. Overview of Findings

The findings were articulated through three themes and eleven subthemes that encapsulate the key elements pertinent to the study objectives (Table 3). The IC’s role depends on the individual profile, expectations, range of responsibilities, and complexity of the illness. While ICs have been recognized as key stakeholders in home-based PC, several barriers and enablers of good-quality palliative home care provision were identified. These findings are discussed in more detail in the following sections and are supported by excerpts from the focus group conversations.

#### 3.2.1. Theme 1: IC Needs and Motivations for Providing Care

In all focus groups, participants reported a set of attributes that determine the needs of ICs and the motivations for providing care at home. Three subthemes were established in this main theme: (a) the heterogeneity of the IC profile, (b) the complexity of illness trajectories, and (c) care as a sense of duty.

(a) The heterogeneity of the IC profile

The participants addressed the heterogeneity of the IC profile in the different contexts in which they carry out their activity and which determine the need for a permanent adjustment of their intervention. This heterogeneity is evident in terms of age, sex, socioeconomic conditions, health status, the scope of tasks and the type of family relationships.

The profile of the IC is predominantly female and may be young (assuming the role of caregiver in the productive phase of their lives) or older (providing care for other family members, often also old). The latter face challenges inherent to the aging process in terms of strength and mental capacity for the care process.

*FGD 1: We currently also have a population of very old caregivers, as is typical, but we also have many caregivers, wives, husbands, and young caregivers as well. The problem is that, in addition to taking care of the family, they also work to maintain the family structure.*

*FGD 2: The majority are older women, although in the last year the age range of our users has dropped considerably and we have noticed that we often have/continue to have women, but many of them are still actively working, which was not common, but the majority are older women with health problems, and physical limitations to care.*

*FGD 4: The type of caregivers and age range is very versatile, also because the ages and stages of the disease of the people we treat here are also very varied. We have very young people who are spouses, and very old people who are also the spouse, but they are two completely different types of spouses. (…). Typically, younger people have more knowledge while older people have physical limitations that sometimes prevent effective care.*

At the same time, the economic conditions of families were highlighted. Some ICs possess many economic assets that enable the acquisition of resources to support care, while a considerable number of ICs face many economic limitations that affect access to basic products, housing conditions and the acquisition of medication.

*FGD 3: It’s interesting. Both in wealthier social classes and in social classes that have nothing, it is impressive how they manage to even take care of themselves. I think it varies. In economic terms, we find everything. It’s not even possible to define a population. (…) Here in my city we have everything, like everywhere else in the world, right? There are very poor people, who don’t have money for basic needs, and there are people who live very well, and it’s different.*

The dominant pattern of family relationships was also valued, with emphasis on marital or kinship relationships (parents and children).

*FGD 1: And our caregivers are characterized as being mostly wives or daughters.*

*FGD 3: No, it varies. When there is a more advanced age, it is the wife or husband who is taking care of the spouse, most of the time, the children accompany them.*

Overall, the IC profile determines the need for professionals to permanently adjust in terms of the education, training and qualification of ICs in view of the transition to the role of IC. Participants report that this transition entails changes in life processes with alterations at the family, work, economic, and leisure levels and in their health and emotional condition and increased overload.

*FGD 3: Being a caregiver is demanding, it is not enough to know a lot, they need to assume care as a part of their own lives, with all the implications that this entails.*

*FGD 1: Many caregivers are at the limit of their abilities. They want to care but the role is very complex in terms of individual, family and social issues.*

(b) The complexity of illness trajectories

The trajectory experienced by ICs is determined by the progression of the disease, as well as its complexity. This means that the greater the complexity of the disease, the greater the number of implications on the physical and/or mental capacity of the sick person, which can lead to greater dependence on others. In this sense, disease trajectories are an aspect that directly influences the transition to the IC role. Participants highlighted that ICs had some difficulties and resistance in adapting to the deterioration of the clinical condition of the ill relative.

*FGD 3: One of the points where informal caregivers have difficulty in adapting to their role has to do with the progression of the disease. Many patients arrive with their autonomy still preserved, in the vast majority, and therefore are able to carry out all their basic activities, and the informal caregiver begins by being more of a companion than a person actively helping with dependency. As the disease progresses, with the worsening of dependence, more and more work and dedication are required from the informal caregiver, and this is where the difficulties and doubts begin.*

As the disease progresses, new challenges arise for ICs in family care. One of the most impactful challenges concerns nutrition and hydration. This theme was consensual in all FGDs, being highlighted as a very sensitive topic for the ICs, as they assume that not feeding the person in their care is synonymous with not providing adequate care, which generates fear and distress.

*FGD 1: One of the concerns, especially in the last (imperceptible) ones, which I notice is mainly food. People consider the fact that the patient is not eating as a failure in the provision of care.*

*FGD 4: I would say it is food and hydration. It often causes confusion for caregivers, whether because they have a reduced appetite and are not eating enough, or because they are not drinking enough water, it causes more confusion for families, realizing that perhaps it no longer makes sense.*

*FGD 3: Food, which is basically the main focus of the family, is the fact that the family member does not eat the way they used to.*

*FGD 2: Questions regarding nutrition and hydration, it’s cultural and it’s important.*

PC teams are aware of this phenomenon and provide teaching on why people in PC do not eat. However, they recognize that sometimes ICs remain reluctant despite clarifications.

*FGD 1: The food, here it is. I think the most important thing about diet is the natural evolution of a serious, progressive and irreversible disease. All areas, lack of strength, dependence, the ability to take care of oneself, decreased appetite, “if your family member eats less, it’s not because he doesn’t want to eat, it’s not because he wants to, it has to do with the natural progression of the disease, and it’s not because he’s eating less that he’s getting worse. […] but what I feel above all is this concern, and that they try to normalize with our culture of taking care of our diet. We are sick and we need a little soup to make us feel better, and especially these older people, who say, “if you are sick, I brought you a little soup to see if you feel better”.*

PC professionals refer to the importance of education in these situations to dispel pre-existing stereotypes and myths, especially among older ICs. Another challenge that influences the role of ICs, as the disease progresses, is the final phase of life. This phase is considered the one with greatest impact on ICs, and the last days and hours of life are those that cause the most challenges for ICs, whether due to the fear of dealing with death or the difficulty in managing symptoms at home.

*FGD 1: At a very advanced stage in which the patient will be with us for a very short time, and therefore have more difficulty in this area of accepting the rapid progression of the situation (…) this issue of care until the end, the issue of dying at home, and sometimes it is this fear of death, (…) but sometimes it is very common for them to ask these questions of “if I die at night, what do I do?”, right? This fear.*

*FGD 4: There is fear of not being able to control the symptoms at home when they decompensate. Soon they are unable to respond to the needs of the palliative patient.*

As a result of difficult-to-manage situations, a constellation of reactions/responses arises, such as ambivalence, insecurity, anguish, anxiety and suffering.

*FGD 3: Often, the perception we have is that the caregiver feels a lot of doubts, above all, they always have a lot of doubts, they ask themselves a lot of questions, because the unknown ends up being just that, the progression of the disease ends up causing them a lot of discomfort and in that sense they always have a lot of doubts. (…) I think it’s anxiety and anguish. Sometimes even in small things, because they want to validate, deep down, if they are doing well. And what they are doing well, if it is good for the family. Above all, I realize this more. If they are doing everything or anything they can do.*

ICs often end up projecting their own suffering onto the patient.

*FGD 1: He tells me he is going to die, he is suffering a lot or when he is in a more advanced stage “he is in great suffering, he has that noise”, and deep down it is the caregiver, no matter how much he explains it, it is the caregiver who is suffering.*

*FGD 2: Essentially, I think it starts with a great difficulty in managing emotions, particularly in managing ambivalence and the feeling that you will be able to take care of someone and the feeling of helplessness in the face of what could or is happening or could happen in the future.*

The difficulty in managing these less positive reactions/responses contributes to IC vulnerability, particularly in terms of self-care. Participants report that most ICs do not have self-care behaviors, either due to lack of time or lack of strategies to promote self-care. To facilitate the experience and respective transition to the role of IC, their needs need to be met, through humanized social and health responses, sensitive to the needs of IC.

*FGD 1: We were talking about emotional issues, I think that caregivers make a great effort to represent and inhibit what they feel, and there is a need to express these emotions, there is also a need to take care of themselves and often they do not have much of this perception. We are the ones who have it and it is not easy to pass this on to them.*

*FGD 2: I have several caregivers who go to the appointment and say: “my husband is the one who is sick, but my life is the one that has stopped”. They may even be people with children and grandchildren, and they feel loneliness and hide in a shell. This is also a part of the profile, because those who are true caregivers, in the true sense of the word, and who care, first think about the other person and then think about how they are going to resolve this issue. (…) Therefore, caring for others is only possible if we are also the best we can be. There is often resistance on the part of caregivers to accept our help. In terms of adaptive strategies, we also try to promote and raise awareness among caregivers about emotional self-care, so that people understand how to maintain it as well.*

For this reason, PC teams raise awareness among ICs using physical and digital materials to encourage self-care.

*FGD 1: Stress management, because sometimes there are multiple competing stresses. There could be some exercises, the tool could be a resource. There is a lot about self-care materials, very simple things, or we even leave it to people to see if they have in fact been paying attention to these areas.*

(c) Care as a sense of duty

During the FGDs, participants reported that ICs have an enormous sense of moral duty, accepting the role of ICs based on feelings of love and reciprocity towards their sick family members. Thus, the motivation for care and the willingness of family members to adopt and continue in the carer role is determined by family values (intrinsic motivation).

*FGD 1: Those who really want to care, who really want to be present at that moment, find a way to be present (…).*

*FGD 4: The caregivers want to care, to make this sacrifice, which for them is not really a sacrifice. It is as if it were a moral obligation, as a family, to do so.*

However, the sense of duty exposes the IC to great social isolation, given that, in most cases, there is only one caregiver who provides care 24 h a day, 7 days a week.

*FGD 4: We forget that the whole day, the whole night, in which this caregiver is normally alone, having to take care of the patient.*

*FGD 2: Therefore, there ends up being a lot of this additional overload on that single caregiver.*

At the same time, many ICs accumulate several roles within the family environment: IC, woman, wife, and mother, among others. Balancing the responsibilities and functions associated with these roles, in order to maintain family functioning, becomes a challenge. Given this imbalance, tension sometimes arises in the management of roles and ambivalence of feelings to the point where ICs raise the possibility of ending their carer role due to an inability to maintain the different roles, leading to the institutionalization of the ill person as the alternative solution.

*FGD 1: We have several caregivers who have to provide for their families, because usually, in the case of daughters, they already have another family with children, grandchildren. They have to continue to provide for their families and at the same time be there for their sick parents.*

*FGD 4: People try to do the best they can and feel that they cannot meet their needs. On the other hand, they want to keep their family members at home too and they have this difficulty in being able to meet their needs and then they live in a dilemma, right? Between seeking institutionalization, or hospitalization, or whatever, to be able to receive better care, versus having it at home, which was a place they would like.*

In sum, there are several determinants that influence the needs of ICs and their preparation for the role of IC, highlighting the heterogeneity of the IC profile, the complexity of the illness trajectories, and the perspective of care as a moral duty. Therefore, professionals need to be attentive and invest in PC literacy.

#### 3.2.2. Theme 2: Perceived Barriers to Good-Quality Palliative Home Care

This theme highlights the main barriers to good quality palliative home care that increase the risk of fragmented and disconnected care. This theme appears anchored in four subthemes, namely (a) limited access and late referral to specialized PC; (b) a lack of social responses; (c) the wear and exhaustion of ICs; and finally, (d) low literacy in PC.

(a) Limited access and late referral to specialized PC

The FGD participants were unanimous in considering that the process of referring and forwarding people to PC is very late, i.e., people have access to specialized PC when they are already in advanced stages of the disease. However, there are many patients with palliative needs who do not receive any type of specialized support. In this way, the complex management of home care processes is aggravated, causing suffering and hopelessness in patients and their ICs.

*FGD 2: We are aware that there are many patients with palliative care needs who are not being followed by palliative care teams. There is a lot of hidden suffering, from all those who need us but who cannot access us.*

*FGD 4: People often come here with patients with terminal illnesses who have no idea what is going on. The diagnosis was made a long time ago and the prognosis has been reserved for a long time. People are in a terminal phase, and their families are not made aware of this. They are told that they have cancer, they are told that they will undergo treatment, they are never told that this cancer is not reversible, they are never told what that means.*

*FGD 4: And I think that some answers are missing here so that caregivers also feel more capable of having their family members at home.*

Participants also highlighted a series of constraints in PC teams that affect access and referral, as well as a person-centered care. One highlighted constraint is the lack of a permanent telephone contact (24 h).

*FGD 3: For caregivers, after they start to experience all the team’s actions and support, one of the things they always say is, “It’s a shame that this isn’t... how do I do it on the weekend, how do I do it at night”, and if you were also available it would be different, and there are times when we realize that (...) They need to feel safe and know that there is someone on the other side who will answer them. For example, on the weekend.*

*FGD 4: We provide support, we manage, having telephone lines that are open from 9 am to 6 pm. However, those who work in palliative care know that the worst times are at night, and at night is when we have fewer resources. Therefore, we have to stop this phase of voluntarism of staying late to work, and we seriously need to invest in the formalization of this continuous support.*

FGDs also highlighted that the shortage of community support teams in PC and the lack of human resources specialized in PC are determining factors that limit access to and the monitoring of patients and families.

*FGD 3: We lack community teams because we could have patients being cared for at home, and with the possibility of dying at home. These teams would be able to support families over a longer period of time. We often see the anguish of family members who have to deal with a patient who is dying at home in their final days, and the support we can provide is only over the phone. We often feel like going home to help. That’s where we have to act…*

(b) Lack of social responses

Based on the analysis of the FG interviews, the available social responses to meet the needs of the person in a palliative situation and their ICs are very limited. In addition to the lack of social support structures, the availability of support products and technical aids (e.g., articulated beds, wheelchairs and diapers) needed to facilitate care at home is also lacking.

*FGD 2: When we talk about places in shelters for caregivers to rest, there are still very few places.*

*FGD 3: As the disease progresses, support is needed in terms of walkers, wheelchairs, bath chairs and articulated beds that allow them to stay at home with them caring for them. One of the most complex things is the moment when they need an articulated bed and we know it is necessary, but not everyone has the financial capacity to make this acquisition. As we have family members who donate some support materials, we end up taking out loans, but they do not meet our needs.*

*FGD 1: Locally, we have health centers and local associations that have technical assistance banks that lend equipment and support products, this helps with this immediate need.*

It was also highlighted that access to social vacancies to support ICs, acquiring caregiver status, as well as the availability of support products, end up being very bureaucratic and time-consuming processes, which are not compatible with the pressing needs of patients and ICs.

*FGD 1: Social security vacancies (state support system) are bureaucratic processes, which limits the response capacity. They end up being an unviable alternative, it ends up being like that.*

*FGD 2: The system is extremely bureaucratic. When the responses arrive, the patients have already died.*

(c) The wear and exhaustion of ICs

From the participants’ perspective, being an IC implies a high expenditure of energy and time and consequent overload, capable of generating negative consequences for their health and well-being. In this sense, professionals reinforce the need to evaluate and prevent risk situations.

*FGD 4: They arrive at a stage of exhaustion, because there were no answers that helped them. And often this is what we try to do in consultations, especially in the first consultations, to make them aware that there is a way to support them so that they do not reach a state of exhaustion that later prevents them from continuing to care for the child and from having their family member at home, which is their main desire.*

*FGD 3: As we were saying earlier, often when they arrive here, they have that feeling of helplessness, of frustration, because they think that no matter what they do, they will not be able to reverse the situation.*

Several causes of burnout and exhaustion were identified, with emphasis on the removal of ICs from support networks due to their exclusivity for care tasks. Often, it is only in cases of extreme overload or when patients are getting worse that ICs resignedly decide to place their family member in short-term hospitalization.

*FGD 2: Caregivers are held hostage by care tasks and no longer have a social life. This leads to tiredness and exhaustion. But when they need help, or when patients have uncontrolled symptoms, they are very resistant to short-term hospitalizations, for example.*

*FGD 3: Whenever we identify the caregiver’s need for rest, we find that it is not an easy process for the caregiver to decide on hospitalization because they have a very strong connection with the patient because they are caring for them 24 h a day.*

It was also mentioned that many ICs feel abandoned by professionals whenever they see a simplification of care in terms of medication and food. There is a false perception that professionals are not doing enough and what is best for their family member, given the lack of care.

*FGD 1: Because there are many interventions throughout the process that bring benefits, and there are many decisions that you make, such as, for example, taking away a medication that the neurologist prescribed three years ago and that was very important at the time, and we suspend that medication, it is a very simple intervention, suspending a medication that is not currently being useful and may even be harming the patient.*

As loss ceases to be a possibility and becomes an inevitability, ICs begin to demonstrate grief responses, which are often maladaptive and lead to anxiety and suffering. Therefore, it is essential to develop strategies that prevent prolonged grief.

*FGD 1: A very important aspect for families is: ‘and when he dies, what will it be like, who will come here? And if it’s at night?’, once again it is information that reduces the uncertainty, fear and exhaustion of caregivers.*

*FGD 3: With overload and exhaustion come the feelings of having failed, of having abandoned, of not having done everything. We must be careful to avoid prolonged mourning and the emergence of mental illness.*

(d) Low literacy in PC

Currently, many ICs do not have the necessary skills to respond to the health demands of their sick family members due to low PC literacy. Given the widespread dissemination of information today, ICs sometimes find contradictory information regarding PC, reinforcing some stereotypes that link PC exclusively to death and end-of-life processes.

*FGD 1: In the past, there was no internet and not so much information. Now there is too much information, and sometimes the excess of information, and especially contradictory information, generates insecurity, instead of empowering and strengthening what the family or the person actually wants to do.*

It was also highlighted that younger ICs have more digital skills for accessing information, while older ICs have more difficulty adapting, given that technologies are not very user-friendly.

*FGD 4: We increasingly find younger people who turn to smartphones very easily to find answers. However, this no longer happens with isolated caregivers, we have to have a different approach.*

*FGD 2: Technologies are good, but for older people it is very limiting. For example, in the simple issuance of an electronic prescription, they do not have the capacity, not even a cell phone, so they can access the code.*

#### 3.2.3. Theme 3: Perceived Enablers of Good-Quality Palliative Home Care

According to the findings obtained, four subthemes were identified: (a) formal and informal support networks; (b) the quality of communication and relationship with community teams in PC; (c) respect for preferences and wishes; and (d) the systematization of the informal caregiver training process.

(a) Formal and Informal support networks

Professionals report that the formal and informal support network is seen as a facilitating factor, mitigating the needs of ICs. An informal support network that includes neighbors, friends, associations and the community, provides the IC with greater proximity and direct support.

*FGD 4: The factor that makes us feel more at ease when we send someone back home is, in addition to this caregiver, knowing if he or she has family living nearby, neighbors who can provide support.*

*FGD 2: In more rural places, this still happens and people who live nearby are available to help each other. The neighbors will play this role. In large cities, there is disorientation given that there is no such available informal support network.*

Regarding formal support networks, the participants emphasize that in urban areas, services and support are more diverse and specialized compared to rural areas. There is clear geographical asymmetry in terms of the distribution of community support teams in PC, public or private care providers, and home service provision associations.

*FGD 1: It is clear that formal resources are more common at the urban level, such as health centers, palliative care teams, and local associations. The same is not true in more rural and remote areas.*

*FGD 4: In the ULS (Local Health Unit), we all work in a network, which allows us to make the best use of resources. When we have patients who live far from our PC team, we try to work with community units to support caregivers with their difficulties, manage symptoms, and make therapeutic adjustments.*

(b) The quality of bonds with community teams in PC

The participants emphasize the relationship of trust and close communication that community support teams establish with ICs, which translate into the availability and empathetic listening offered by professionals.

*FGD 1: In the monitoring processes, we have to develop adequate communication, make information available, and whether in person or remotely we try to listen and be assertive and this generates a feeling of security in the caregiver, that they are being understood.*

*FGD 3: Availability. We are available to support them. Even if they don’t need it, they know that they have someone available to help.*

ICs often praise the support provided by community teams for the safe way in which they are informed and clarified by professionals from PC teams, allowing them to anticipate numerous crisis situations, especially in symptom management, end-of-life care and bereavement.

*FGD 4: Above all, we try to emancipate them, empower them, that is, anticipate certain issues that at that time may not be a priority, but that in the future may become necessary.*

*FGD 2: It is a basic concern for us to develop practices that are preventive, whether in controlling symptoms, in the process of death and loss.*

(c) Respect for preferences and wishes

Home is the preferred place of care for ICs, as it favors comfort and the centrality of care for the person, in addition to respecting the wishes and desires of patients.

*FGD 4: Normally, we question what the preferred place for care and death is, to better manage expectations.*

*FGD 2: Caregivers suffer because they are unable to achieve the goal of keeping their family member at home until they die. This is often the objective they have in mind.*

Respect for preferences and wishes are seen as practices that promote the quality of PC, with the achievement of greater comfort, QoL, and dignity in the care provided.

*FGD 3: Allowing the person to be cared for at home creates a greater connection with significant others and facilitates access to memories while respecting their wishes and desires. Not to mention that patients do not need to travel, which for a person confined to bed, having to be transported by ambulance causes discomfort. Caring at home with the support of a specialized team makes all the difference in terms of a better quality of life and preservation of dignity.*

(d) The systematization of the informal caregiver training process

The participants believe that the IC training process should be planned and integrated into the care plan and should not depend on chance.

*FGD 1: There must be a well-designed, well-defined plan, in which the organizational methodologies include the training of caregivers. After identifying the informal caregiver, it is important to assess their conditions for assuming this role and to understand the entire family process in depth. The initial step is to explore whether the person wants to be an IC and whether there is an assumed role or not, or whether they want to assume it, and then, if it is assumed, what the person needs and what the family needs.*

Another strategy identified by the interviewees involves the safe transition of care, wherein training ICs is a fundamental requirement to guarantee the quality of care and the satisfaction of patients and their families.

*FGD 1: Whenever a patient changes service or team, it is essential to ensure continuity of care between health responses and social responses.*

Finally, the need to expand education modalities from a knowledge-centered approach to a learning-centered approach was also highlighted, where training ICs to use digital resources can facilitate the resolution of immediate problems (e.g., exchanging messages via WhatsApp).

*FGD 2: For a patient who is at home 40 km from the PC team, (…) we resort to exchanging messages via WhatsApp and teleconsultation, and this greatly helps with the comfort of the patient and family.*

*FGD 4: Whether by telephone or in-person consultation, I think these end up being the most effective means and the ones they trust the most.*

*FGD 3: We see older people who have already mastered technologies and do things. So, I think they effectively learn to use WhatsApp, for example, to talk to their grandchildren. In other words, I think it is an added value that expands communication.*

Although there may be limitations to the use of digital resources, their use improves and expands the ability of ICs to connect with PC professionals while providing care at home.

## 4. Discussion

The healthcare professionals in this study highlighted how to enhance ICs’ perceived preparedness and alleviate their caregiving load by prioritizing education, information dissemination, and fostering strong collaborative relationships between families and PC teams. This study offers valuable insights into home-based PC interventions and the essential elements for high-quality PC.

Multiple variables determine IC preparedness to deliver PC at home. Among them, family values surfaced as a significant motivation that supported the readiness of ICs to assume and persist in the caregiving role [51]. These values collectively enabled family members to sustain their commitment to caregiving and offered the necessary motivation to address the many obstacles inherent in caregiving [27,52]. Often, ICs expressed experiencing coercion to assume caring responsibilities and a deficiency of autonomy stemming from societal expectations to care for an ailing relative [53]. Carers with a strong inclination to support the patient described a heightened sense of perceived readiness for their position, but those swayed by societal standards often expressed apprehension [27]. The study participants identified age and IC relationships with the ill person as demanding, as this may impact their individual needs and burdens. The majority of ICs are female, daughters and/or wives as the role of care has historically been seen to be inherent in women [54]. Additionally, most ICs are older, exposing them to physical and psychological health difficulties during end-of-life care [55]. One of the most frequent challenges for ICs in family care is related to the need for hydration/nutrition, as well as dealing with death at home [56]. The heterogeneity in IC profiles is reflected in differing expectations of participation, since ICs also have varied health and socioeconomic conditions when caring for their loved ones [57].

The findings also emphasize a set of barriers that limit high-quality PC at home, namely limited access to PC, the shortage of social responses, IC burnout and exhaustion, and poor PC literacy. These elements are important barriers already indicated by the available evidence [12]. Other constraints in PC teams were mentioned: the lack of a permanent telephone line/contact in PC; the shortage of specialized PC support teams; the lack of resources, time, and responses; and the bureaucratization of social processes. These were considered constraints that do not promote home care focused on the needs of the IC and people in palliative situations [58,59]. According to the literature, the main difficulties of ICs include a feeling of disorientation and abandonment due to lack of support from health teams, social isolation, emotional overload, the absence of a perception of the limit of exhaustion, anticipatory grief, and the lack of digital literacy in health and PC [12,58,60,61]. These barriers profoundly affect the possibility of having good PC with an impact on patient and IC QoL at home. The lack of adequate support, emotional overload and scarcity of both material and human resources generate a cycle of exhaustion and helplessness for ICs [56,62]. At the same time, improving access to information and ongoing support, as well as training professionals in PC, are essential to mitigate these obstacles [4,63].

They were also facilitators of high-quality PC at home, particularly the importance of formal and informal support networks [5,61,64]. According to the findings, ICs value the availability, empathy and quality of communication of specialized PC teams, which reinforce relationships of trust [63]. The personalized and prophylactic work carried out by PC professionals was also highlighted, thus preventing suffering [65]. The preference for the home as a place of care is respected and its advantages in the QoL of the person in a palliative situation are praised [13,26,66]. Healthcare professionals also articulated the necessity to address the preferences of patients and ICs. In this context, selecting the home as the preferable setting for care and end of life, as it enables proximity to and the safeguarding of loved ones, provides a secure and familiar environment where patients can exercise autonomy and address care needs [67]. Assisting the patients and ICs as a cohesive unit is essential for delivering high-quality PC at home. This necessitates education, training and support for patients and their carers regarding expectations as functionality diminishes and illness advances. This also entails clarity regarding care expectations and service constraints. PC patients and their ICs can be equipped with knowledge and, if they choose, become active participants in the provision of care. Furthermore, assisting families requires providing sufficient IC respite and aid with caregiving responsibilities. In this regard, Klarare et al. [68] stated that individuals with cancer expressed a desire for their participation to be acknowledged by healthcare professionals and integrated into care-related decisions. Being empathic, adopting a person-centered approach, and showing concern for the well-being of patients and their ICs were revealed by our findings. Professionals identified these attributes as essential for fostering IC trust in healthcare professionals and affirmed their role as part of a collaborative team dedicated to enhancing the patient’s well-being [52,68]. A bond of trust with PC staff typically develops throughout the course of an illness. Previous studies have shown the significance of integrating empathy into care, particularly in home PC, as a crucial element of professional competence that fosters enduring relationships with patients and their families [69,70,71]. Moreover, a meta-ethnographic study indicates that 24 h professional care is a crucial element in enhancing the care experience, corroborating our findings that emphasize the significance of skilled, communicative, and supportive formal PC in assisting IC preparedness to face home caregiving situations [70,72].

These essential elements align with the overarching conclusions of previous worldwide studies and the components of optimal PC practices implemented in many nations. These features are empirically grounded in the comprehensive analysis of established successful palliative home care programs, together with the perspectives of both users and providers of these services. Establishing and funding palliative home care programs that incorporate these features is vital for facilitating a favorable experience for patients and ICs at the EoL. Further research should also include IC voices to provide insights into their experiences and inform caregiving practices in the home setting. Likewise, our study highlights the necessity for research that examines the internal, individual, context-specific experiences of informal caregiving while also addressing the moral and ethical dimensions of caregiving motivations [73,74], given that the availability and continuity of informal caregiving are essential for supporting strained formal services globally.

### 4.1. Strengths and Limitations

Our study has several strengths and limitations. One strength is the integration of the subjective perspectives of professionals through FGDs, providing a thorough and nuanced understanding of their critical considerations in PC. Additionally, this study was conducted in accordance with established criteria for focus group interviews [42] and the COREQ guidelines [43]. The analysis was meticulously documented and repeatedly discussed among the researchers at each stage. To enhance the study’s credibility, the researchers sought to maintain reflexivity throughout each phase of the research process. Although we employed a purposive sample of 34 professionals, we deemed the sample adequate as we discerned variances in their experiences and attained data saturation following the focus group interviews.

A potential shortcoming of the current study may be the inclusion of professionals who were previously acquainted. Utilizing pre-existing groups allows for the observation of certain facets of their interactions, thereby approximating naturally occurring data, akin to that gathered through participant observation. A further benefit of this technique is that professionals might correlate each other’s remarks with specific occurrences in their mutual professional experiences. They could confront one another with discrepancies between their stated beliefs and their real behavior [42].

Another limitation might be that the interview questions were not pilot-tested. We also recognize that PC configurations and work practices vary by country and across various global regions. Variations in participant staffing methods, professional education in PC, and decision-making structures may restrict the applicability of our findings. Focus groups are prone to bias as dominant participants or the moderator can influence group and individual perspectives; hence, it is possible that certain experiences may remain unexpressed. The biases of the moderator and assistant moderator affected both the inquiries posed and the subsequent analysis. Certain participants may have been reluctant and conveyed their experiences in alignment with the expectations of others. Nonetheless, we contend that the participants felt motivated to communicate and deliberate on their experiences within the group. Furthermore, the participants were permitted to provide comments on the moderator’s comprehension of the essential insights derived from the FGDs. During data collection, we utilized precise question formulation, established rapport with each participant, and ensured that data gathering occurred simultaneously with data analysis. We sought to reconcile adherence to the themes in the data, crucial for generating insight, with an awareness of inevitable prejudices, necessitating reflexivity.

Another study weakness is that the ratio of male to female professionals was unbalanced; this reflects the trend towards more women in the Portuguese healthcare system [75]. Furthermore, most participants were nurses because these professionals are the most numerous in the PC teams, dedicating significant time to direct assistance to patients and their families. Also, this study did not integrate professionals from other regions (e.g., northern and island regions) or countries, which may limit the transferability of the research findings.

### 4.2. Implications for Practice

Proper attention to ICs is crucial as they play a vital role in patient care; if an IC is experiencing distress, they may fail to offer emotional support or be fully there for their ailing cared recipient. Consequently, it is essential to comprehensively incorporate ICs in therapeutic interactions and to avert their potential caregiving overload and burden. Enhanced inter-professional communication and coordination, alongside less bureaucracy and more transparent methods for acquiring resources and services, will diminish the necessity for patients and their ICs to remain attentive to ensure quality care. Specifically, individuals who exhibit lower assertiveness, lack familial support, or possess little health literacy will derive advantages from this. While there is an expanding corpus of studies delineating the essential factors in delivering quality palliative home care, there is also a necessity for investigations to ascertain the attributes of practitioners, home environments, and broader support systems that enable effective care.

Preparedness and motivations for providing care may vary over time depending on the care recipient’s condition and the types of care tasks [73]. This means that ICs may experience feelings of reciprocity and commitment, along with burden and stress, possibly due to an over-investment in their care role [73]. The ambiguity surrounding the effects of caregiving willingness—whether beneficial or detrimental—highlights the need for customized interventions to support ICs at home. This conclusion may illustrate the temporal and dynamic nature of the caregiving journey, enhancing our understanding of the readiness to provide informal care as a process that needs ongoing support from PC teams. Furthermore, there is a prevailing taboo associated with the willingness to provide care at home, because reluctance to do so could be deemed socially unacceptable.

## 5. Conclusions

To the best of our knowledge, this is one of the first attempts to explore challenges in the delivery of informal home-based PC in Portugal through the lens of professionals who work in PC. Three overarching themes emerged: (1) IC needs and motivations for providing care; (2) perceived barriers to good-quality palliative home care; and (3) perceived enablers of good-quality palliative home care. The essential elements of quality PC should be standardized, but how to achieve them should be flexible as solutions are derived from the local context and local providers. Given the pivotal role of ICs, this study highlights the significant benefits of home-based PC in improving IC satisfaction and increasing the likelihood of patients dying at home. Moreover, well-trained ICs diminish the incidence of healthcare utilization, especially emergency department visits and hospital entrances. Assistance should be enhanced by staff and health services, demonstrating skilled knowledge and the prioritization of the needs and preparedness of ICs, who are both targets and partners of care. In this vein, healthcare services and care practitioners should refine their communication strategies, actively foster meaningful inclusion, address access challenges, and augment the support offered to patients and ICs.

## Figures and Tables

**Table 1:** Six-phase process of reflexive thematic analysis (based on Braun and Clarke [47]).

Analytic Phases	Description
1. **Familiarizing with the data**	This phase involves a comprehensive analysis and evaluation of transcripts.
2. **Generating initial codes**	The codes are allocated to concepts pertinent to the research question.
3. **Searching for themes**	The codes are classified, and themes emerge from the data.
4. **Reviewing themes**	At this stage, the themes are reviewed by the research team.
5. **Defining and naming the theme**	At this point, the researcher provides an exhaustive analysis of the thematic framework. The themes are identified, outlining a relationship among them.
6. **Producing the report**	The themes are developed and conveyed in a report format.

**Table 2:** Description of participants (*n* = 34).

		FGD 1	FGD 2	FGD 3	FGD 4
**Number of Participants**		12	8	7	7
**Age (Mean ± SD)**		42.3 (10.9)	41.3 (9.53)	45.3 (8.32)	35.6 (6.49)
**Sex**	Female	10	8	5	7
	Male	2	0	2	0
**Profession**	Nurse	6	6	5	5
	Doctor	2	1	2	1
	Social Worker	1	0	0	1
	Psychologist	3	1	0	0
**Advanced training in PC**		8	5	4	2

**Table 3:** Main themes and subthemes.

Main Themes	Subthemes
IC needs and motivations for providing care	(a) The heterogeneity of the IC profile
	(b) The complexity of illness trajectories
	(c) Care as a sense of duty
Perceived barriers to good-quality palliative home care	(a) Limited access and late referral to specialized PC
	(b) A lack of social responses
	(c) The wear and exhaustion of ICs
	(d) Poor literacy in PC
Perceived enablers of good-quality palliative home care	(a) Formal and informal support networks
	(b) The quality of bonds with community teams in PC
	(c) Respect for preferences and wishes
	(d) The systematization of the IC training process

## Data Availability

All data generated or analyzed during this study are included in this article. This article is based on the first author’s master’s dissertation in Palliative Care at the School of Health Sciences—the Polytechnic University of Leiria (supervised by Carlos Laranjeira).

## Supplementary Materials

The following supporting information can be downloaded at: https://www.mdpi.com/article/10.3390/healthcare13090978/s1, Table S1: Consolidated criteria for reporting qualitative studies (COREQ): 32-item checklist.
